# Development and validation of machine learning models for predicting STAS in stage I lung adenocarcinoma with part-solid and solid nodules: a two-center study

**DOI:** 10.3389/fonc.2025.1682633

**Published:** 2025-10-29

**Authors:** Qing-Lin Ren, Liu Lin, Kai Chu, Xin-Rong Xu, Hui-Jun Wang, Jun Wu, Jin-Zhi You, Jun-Xi Hu, Xiao-Lin Wang, Yu-Sheng Shu

**Affiliations:** ^1^ Department of Graduate School, Dalian Medical University, Dalian, China; ^2^ Department of Thoracic Surgery, Wuxi People’s Hospital, Wuxi, China; ^3^ Department of Graduate School, Xuzhou Medical University, Xuzhou, China; ^4^ Department of Thoracic Surgery, Northern Jiangsu People’s Hospital, Yangzhou, China; ^5^ Department of Thoracic Surgery, The Affiliated Suqian Hospital of Xuzhou Medical University, Suqian, China

**Keywords:** spread through air spaces, lung adenocarcinoma, machine learning, solid and part solid component, surgical strategy

## Abstract

**Background:**

This study aimed to preoperatively predict spread through air spaces (STAS) in stage I lung adenocarcinoma presenting as part-solid and solid nodules by leveraging clinical features and machine learning models, thereby guiding surgical decision-making and enhancing patient counseling.

**Methods:**

A total of 473 patients were retrospectively enrolled, including 353 from our center and 120 from an validation cohort. Predictive features were selected using maximum relevance minimum redundancy (mRMR) and least absolute shrinkage and selection operator (LASSO) algorithms. Seven machine learning models—logistic regression, random forest, support vector machine (SVM), extreme gradient boosting (XGBoost), adaptive boosting (AdaBoost), light gradient boosting machine (LightGBM), and category boosting (CatBoost)—were developed and evaluated using receiver operating characteristic curves, calibration plots, and decision curve analysis (DCA). Feature importance was assessed using Shapley Additive Explanations (SHAP). A web-based nomogram was constructed for clinical application.

**Result:**

STAS was present in 44.76% of the training set and 50.83% of the validation cohort. Seven predictors were selected to construct the predictive models. The XGBoost model demonstrated superior performance with an AUC of 0.889 (95% CI, 0.852–0.926) in training and 0.856 (95% CI, 0.789–0.928) in validation. The calibration curves in training and validation set exhibited good agreement between the predictions and actual observations. The Decision Curve Analyses (DCA) provide significant clinical utility. SHAP analysis identified the most important predictors for STAS as CEA, vascular convergence, proGRP, age, AFP, smoking history, and CTR.

**Conclusion:**

The XGBoost model provides robust preoperative prediction of STAS and may assist clinicians in optimizing surgical strategies for patients with stage I lung adenocarcinoma.

## Introduction

Lung cancer remains one of the most commonly diagnosed malignancies and the leading cause of cancer-related mortality globally. In China, it accounts for approximately ​1.06 million new cases and 0.73 million deaths annually (1). Lung adenocarcinoma (LUAD) is the predominant histological subtype, comprising approximately 85% of non-small cell lung cancer (NSCLC) cases (2, 3). For stage I NSCLC patients undergoing curative R0 resection, 5-year recurrence-free survival (RFS) and overall survival (OS) range from 62.5%–64.7% and 78.7%–81.9%, respectively (4). Despite achieving negative resection margins, early-stage LUAD patients continue to face high locoregional recurrence rates.

Spread through air spaces (STAS) is a newly recognized form of invasion in lung cancer, first proposed in the 2015 WHO classification. It is defined as micropapillary clusters, solid nests, or single cancer cells infiltrating into air spaces beyond the main tumor edge (5, 6), STAS is an independent predictor of poor outcomes in stage I NSCLC and is associated with increased recurrence and reduced survival (7, 8). It also raises recurrence risk in LUAD patients treated with limited resection (9, 10).

With advances in imaging technology, lung cancer is being detected earlier, and smaller nodules are considered suitable for sublobar resection, providing surgeons with more opportunities to choose this approach. providing surgeons with more opportunities to opt for sublobar resection. However, several studies have shown that, compared to lobectomy, sublobar resection is associated with lower RFS and OS in patients with STAS-positive tumors. Patients who underwent wedge resection had significantly worse RFS and OS than those who underwent lobectomy (11). Thus, lobectomy is associated with better outcomes for STAS-positive T1 LUAD compared to sublobar resection (12, 13). Therefore, accurately selecting the surgical approach preoperatively is critical.

Given the importance of preoperative STAS identification for optimal surgical decision-making, enhancing the accuracy of these predictions is crucial for improving patient outcomes. Machine learning (ML) models have gained significant traction in disease prediction due to their ability to process high-dimensional data efficiently (14, 15). However, further optimization and validation of ML models for preoperative STAS prediction are needed to improve their clinical applicability.

We developed several ML models for preoperative STAS prediction using clinical and radiological data from our institution, followed by external validation in an independent cohort from another medical center. The primary goal of this study is to accurately identify STAS preoperatively, facilitating precise surgical decisions, improving patient prognosis, and providing valuable insights for clinical treatment strategies.

## Materials and methods

### Patients

**Figure 1:**
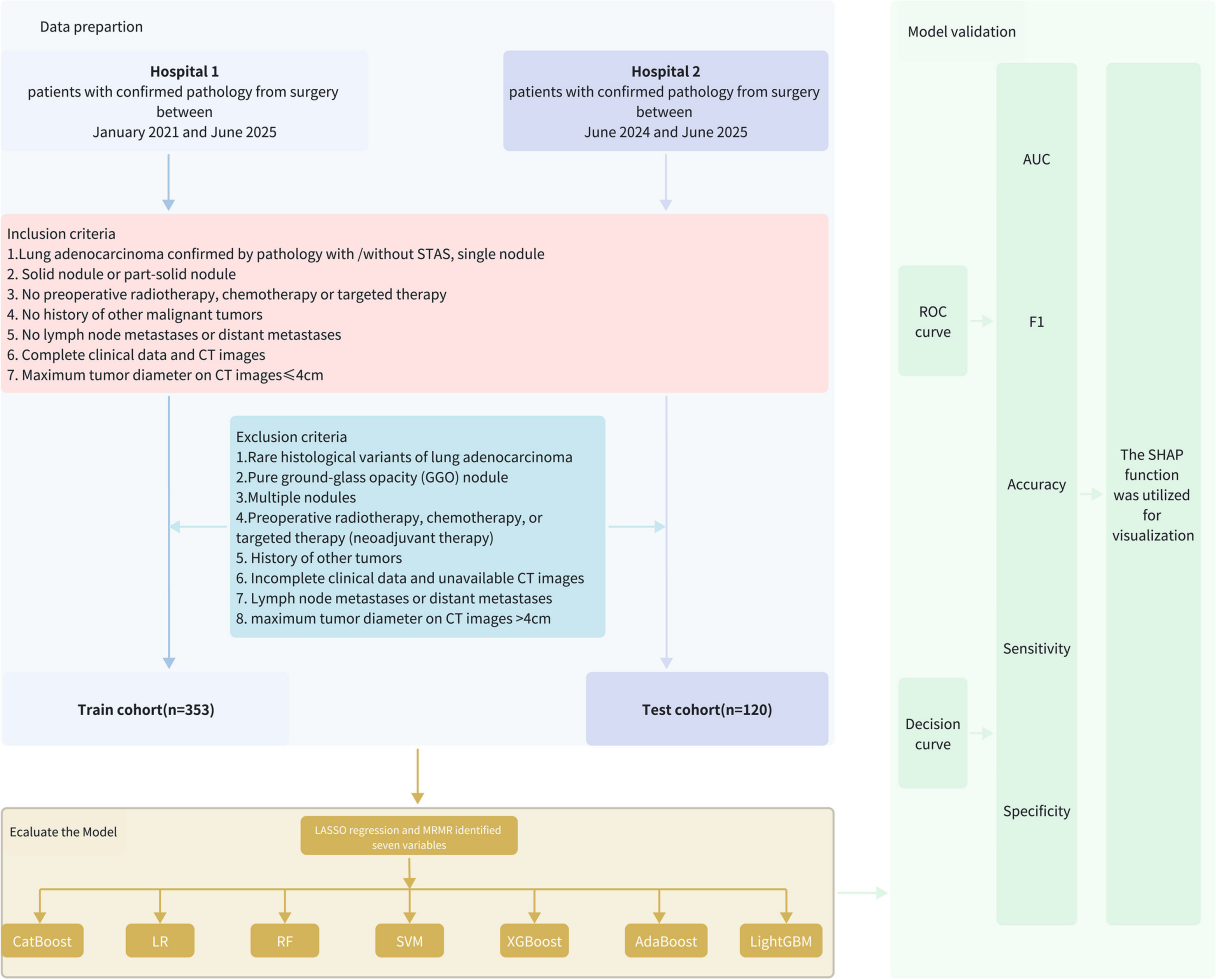
The flow diagram of the study.

Clinical data were collected from 158 cases of stage I lung adenocarcinoma with STAS admitted to Northern Jiangsu People’s Hospital between January 2021 and June 2025. These cases were compared with clinical data from 195 stage I lung adenocarcinoma patients without STAS (the flowchart of this study is shown in **Figure 1**). This study was approved by the Ethics Committee of Northern Jiangsu People’s Hospital.

#### Inclusion criteria

1. Lung adenocarcinoma confirmed by pathology with or without STAS and single nodule.
2. Solid or part-solid nodule.
3. No preoperative radiotherapy, chemotherapy or targeted therapy.
4. No history of other malignant tumors.
5. No lymph node metastases or distant metastases.
6. Complete clinical data and CT images available.
7. Maximum tumor diameter ≤ 4 cm on CT.

#### Exclusion criteria

1. Rare histological variants of lung adenocarcinoma.
2. Pure ground-glass opacity (GGO) nodules.
3. Multiple nodules.
4. Preoperative radiotherapy, chemotherapy, or targeted therapy (neoadjuvant therapy).
5. History of other malignancies.
6. Incomplete clinical data or unavailable CT images.
7. Lymph node metastases or distant metastases.
8. Maximum tumor diameter on CT images >4cm on CT.

Based on the postoperative pathological results, patients were classified as either STAS-positive or STAS-negative.

### Radiological and histological evaluation

Based on findings in the lung window, the solid component was defined as a patch that completely obscures the underlying lung parenchyma and the GGO component was defined as a hazy area of increased lung attenuation with preserved bronchial and vascular margins. The part-solid nodule was defined as a lesion containing both GGO and solid components, solid nodule was defined as a lesion consisting solely of solid components. In histological evaluation, we focused on stage I lung adenocarcinoma with nodules consisting of solid components, excluding pure GGO nodules.

CT features evaluated included pleural invasion, vascular invasion, spiculation, lobulation, vascular convergence, pleural traction, pleural indention, air bronchogram, vacuole, and consolidation/tumor ratio (CTR). CTR was quantified as the ratio of the tumor consolidation diameter to the total diameter. These CT features were defined and assessed according to previous reports (16–19). Two radiologists with over 10 years of chest imaging experience validated the reliability of the radiological assessments. Only features with good inter-observer agreement between the two radiologists were included in subsequent analyses. Both radiologists were blinded to the patients’ STAS status.

### Clinical data collection

Clinical information was obtained through the hospital’s medical record system. The clinicopathologic features included the age at surgery, sex, smoking history, BMI, pathologic tumor(T) stage (seventh edition of the lung cancer staging system) (20), tumor location, operative methods, forced expiratory volume in 1 s (FEV1), and diffusing capacity of the carbon monoxide (DLCO). Laboratory findings on admission included serum levels of white blood cell (WBC), neutrophil, lymphocyte, monocyte, platelet, albumin, neutrophil to lymphocyte ratio (NLR), monocyte to lymphocyte ratio (MLR), platelet to lymphocyte ratio (PLR), carbohydrate antigen 125 (CA125), alpha-Fetoprotein (AFP), carcinoembryonic antigen (CEA), neuron-specific enolase (NSE), cytokeratin 19 Fragment 21-1(cyfra21-1) and pro-Gastrin-Releasing Peptide (proGRP) within 2 weeks prior to surgery. In this study, patients who underwent lobectomy, segmentectomy, or wedge resection were reviewed. Variables with >30% missingness were removed. Likewise, patients whose records exceeded this threshold across candidate predictors were excluded from model development. For the remaining data, missing values were imputed using the mode for categorical variables and the mean for continuous variables.

We aimed to develop a machine learning model to predict the presence of STAS in stage I lung adenocarcinoma patients using preoperative indicators. Thus, operative methods were excluded from the construction of the machine learning model.

### External validation

A validation cohort from Wuxi People’s Hospital, affiliated with Nanjing MedicalFI University, was included for external validation. This cohort included 120 patients, 59 of whom were pathologically confirmed as STAS-positive and 61 as STAS-negative, all meeting the inclusion and exclusion criteria.

### Predictive model construction and evaluation

Maximum relevance minimum redundancy (mRMR) was applied to the initial feature set to reduce data dimensionality (21). Subsequently, the least absolute shrinkage and selection operator (LASSO) logistic regression was employed to identify key features and develop a predictive model for STAS (22).

To reduce dimensionality while retaining non-redundant information, we first applied maximum relevance minimum redundancy (mRMR) using the mRMRe framework (mutual-information–based relevance with redundancy penalization). We used ensemble selection with feature_count = 15 and solution_count = 1, targeting the binary outcome (STAS positive or negative). The retained candidates were then passed to LASSO logistic regression (glmnet) with α = 1. The regularization parameter λ was tuned by 10-fold stratified cross-validation, minimizing binomial deviance. We used λ_min = 0.03469344 for subsequent selection, yielding seven non-zero predictors (CTR, age, smoking history, AFP, CEA, proGRP, vascular convergence).

Seven predictive models were constructed, including logistic regression, random forest, support vector machines (SVM), extreme gradient boosting (XGBoost), adaptive boosting (AdaBoost), light gradient boosting machine (LightGBM), and category boosting (CatBoost) (23–26) and were trained on the selected clinical features using stratified cross-validation (primary metric AUC). Logistic regression (logit link) reported class probabilities without additional penalties. Random Forest used the Gini criterion with 500 trees and CV-tuned mtry (final mtry = 5). SVM (RBF kernel) tuned C and γ (selected: C = 1×10^6^, γ = 1×10^-6^). XGBoost (objective = binary:logistic) tuned learning rate (η), tree depth, min_child_weight, and subsample by 10-fold CV with early stopping, then trained for nrounds = 1000. AdaBoost used mfinal = 10 based on the training-error plateau. LightGBM (metric = AUC) used max_depth = 8 and nrounds = 1000 with leaf/sampling controls chosen by CV. CatBoost (loss = Logloss, eval_metric = AUC) trained with iterations = 1000, learning_rate = 0.1, depth = 8, while l2_leaf_reg and rsm were tuned by CV. After hyperparameter tuning within the training set by stratified 10-fold CV (primary metric AUC), each model was refitted on the full training set and evaluated once on an validation set; we report both cross-validation and external validation performance.

Model performance was evaluated using area under the receiver operating characteristics curve (AUC) and decision curve analysis (DCA) to assess both discriminatory power and clinical utility. Additionally, Shapley additive explanations (SHAP) analysis was employed to interpret model results and assess feature importance, with particular focus on the top-performing machine learning model.

### Statistical methods

Statistical analyses were performed using R software (version 4.3.1). Variables with a missing data rate exceeding 20% and outliers were excluded, with remaining missing values addressed via multiple imputation. Continuous variables following a normal distribution were expressed as mean ± standard deviation (x ± s) and compared using the t-test. For non-normally distributed continuous variables, data were presented as median (25th percentile, 75th percentile) [M (P25, P75)] and analyzed using the Mann-Whitney U test. Categorical variables were summarized as counts and percentages [n (%)] and compared using the chi-square test or Fisher’s exact test, as appropriate.

## Results

### Baseline characteristics of patients

A total of 473 stage I lung adenocarcinoma patients, with either STAS-positive or STAS-negative status, met the eligibility criteria for this study. These patients were divided into the training set (n = 353; data from Northern Jiangsu People’s Hospital) and the independent validation cohort (n = 120; data from Wuxi People’s Hospital, affiliated with Nanjing Medical University).

In the training set, 158 patients were STAS-positive, accounting for 44.76%. In the validation set, 61 patients were STAS-positive, representing 50.83%. Demographic data of all patients were thoroughly examined before modeling.

**Table 1** summarizes the baseline characteristics and perioperative serum variables of the 473 patients. Variables such as age, CEA, CYFRA 21-1, proGRP, NLR, CTR, gender, smoking history, pleural invasion, and vascular convergence demonstrated statistically significant differences between groups in the training set (p< 0.05) but not in the validation set. CEA was the only variable that showed significant differences in both cohorts.

**Table 1:** Characteristics baseline of patients in train set and validation set.

	Train-total (n = 353)	STAS negative (n = 195)	STAS positive (n = 158)	*P* value	Validation -total (n = 120)	STAS negative (n = 59)	STAS positive (n = 61)	*P* value
Age, (years)				0.004				0.426
Mean ± SD	63.13 (10.01)	61.82 (20.30)	64.74 (10.15)		64.18 (9.44)	65.08 (9.35)	63.31 (9.52)	
Gender, No. (%)				0.012				0.102
Female	186 (52.69)	115 (58.97)	71 (44.94)		57 (47.50)	33 (55.93)	24 (39.34)	
male	167 (47.31)	80 (41.03)	87 (55.06)		63 (52.50)	26 (44.07)	37 (60.66)	
BMI (Kg/m^2^)				0.773				0.257
Mean ± SD	25.12 (3.42)	25.21 (3.49)	25.01 (3.33)		23.85 (2.73)	23.62 (2.87)	24.08 (2.59)	
smoke, No. (%)				0.013				0.187
no	244 (69.12)	146 (74.87)	98 (62.03)		69 (57.50)	38 (64.41)	31 (50.82)	
yes	109 (30.88)	49 (25.13)	60 (37.97)		51 (42.50)	21 (35.59)	30 (49.18)	
FEV1, (L)				0.612				0.589
Mean ± SD	2.33 (0.89)	2.29 (0.63)	2.39 (1.13)		2.34 (0.71)	2.30 (0.64)	2.38 (1.08)	
DLCO, (mmol/min/kPa)				0.856				0.785
Mean ± SD	6.04 (1.71)	6.01 (1.74)	6.07 (1.68)		6.12 (1.51)	6.10 (1.68)	6.14 (1.81)	
CE125, (U/ml)				0.761				0.916
Mean ± SD	11.26 (10.47)	10.68 (4.99)	11.97 (14.63)		11.15 (11.46)	9.79 (8.90)	12.48 (13.42)	
AFP, (ng/ml)				0.636				0.364
Mean ± SD	3.01 (2.42)	3.15 (3.05)	2.83 (1.28)		2.90 (1.33)	2.73 (1.08)	3.06 (1.52)	
CEA, (ng/ml)				0.004				0.048
Mean ± SD	3.30 (3.73)	2.83 (2.39)	3.88 (4.86)		3.92 (7.23)	4.38 (7.59)	3.47 (6.88)	
NSE, (ng/ml)				0.266				0.209
Mean ± SD	13.73 (3.76)	13.92 (4.05)	13.49 (3.36)		11.64 (3.66)	11.35 (3.66)	11.93 (3.67)	
cyfra.21.1, (ng/ml)				0.038				0.238
Mean ± SD	2.29 (1.01)	2.19 (1.00)	2.41 (1.02)		2.94 (1.41)	2.90 (1.55)	2.99 (1.27)	
proGRP, (pg/ml)								
Mean ± SD	42.15 (23.98)	39.16 (21.27)	45.83 (26.56)	0.002	41.22 (18.87)	42.24 (23.15)	40.23 (13.65)	0.395
WBC, (×10^9^/L)				0.689				0.283
Mean ± SD	5.97 (1.83)	5.96 (1.77)	5.98 (1.90)		5.84 (1.58)	5.61 (1.33)	6.06 (1.78)	
Neut, (×10^9^/L)				0.347				0.389
Mean ± SD	3.92 (3.44)	4.01 (4.37)	3.81 (1.72)		3.57 (3.04)	3.19 (0.90)	3.94 (4.15)	
Lymphocyte, (×10^9^/L)				0.093				0.313
Mean ± SD	1.69 (0.56)	1.73 (0.52)	1.66 (0.61)		1.84 (0.62)	1.78 (0.62)	1.89 (0.62)	
Monocyte, (× 10^9^/L)				0.571				0.472
Mean ± SD	0.39 (0.20)	0.39 (0.17)	0.40 (0.23)		0.49 (0.19)	0.46 (0.14)	0.51 (0.23)	
Platelet, (× 10^9^/L)				0.594				0.781
Mean ± SD	190.08 (62.55)	193.10 (65.32)	186.35 (58.93)		213.53 (61.84)	212.73 (52.61)	214.31 (70.06)	
Albumin, (g/L)				0.317				0.063
Mean ± SD	45.56 (13.87)	44.68 (4.11)	46.65 (20.21)		39.06 (2.70)	39.53 (2.59)	38.61 (2.75)	
NLR				0.05				0.852
Mean ± SD	2.56 (2.26)	2.49 (2.53)	2.65 (1.89)		2.09 (1.44)	1.96 (0.80)	2.21 (1.86)	
MLR				0.232				0.783
Mean ± SD	0.25 (0.13)	0.24 (0.11)	0.26 (0.14)		0.29 (0.16)	0.28 (0.10)	0.30 (0.20)	
PLR				0.378				0.350
Mean ± SD	120.12 (47.88)	118.19 (46.27)	122.49 (49.84)		127.27 (54.44)	131.45 (55.08)	123.22 (53.96)	
CTR				0.012				0.219
Mean ± SD	0.88 (0.54)	0.84 (0.58)	0.91 (0.48)		0.87 (0.52)	0.84 (0.58)	0.90 (0.48)	
Maximum solid component diameter (cm)				0.214				0.001
	1.75 (1.40)	1.72 (1.32)	1.79 (1.48)		1.82 (1.72)	1.57 (1.64)	2.06 (1.66)	
T, No. (%)				0.333				0.367
T1A	43 (12.18)	28 (14.36)	15 (9.49)		6 (5.00)	5 (8.47)	1 (1.64)	
T1B	203 (57.51)	114 (58.46)	89 (56.33)		46 (38.33)	23 (38.98)	23 (37.70)	
T1C	71 (20.11)	34 (17.44)	37 (23.42)		46 (38.33)	21 (35.59)	25 (40.98)	
T2A	36 (10.20)	19 (9.74)	17 (10.76)		22 (18.33)	10 (16.95)	12 (19.67)	
Pleural invasion, No. (%)				0.028				0.322
no	307 (86.97)	177 (90.77)	130 (82.28)		75 (62.50)	40 (67.80)	35 (57.38)	
yes	46 (13.03)	18 (9.23)	28 (17.72)		45 (37.50)	19 (32.20)	26 (42.62)	
Vascular invasion, No. (%)				0.343				0.013
no	338 (95.75)	189 (96.92)	149 (94.30)		106 (88.33)	57 (96.61)	49 (80.33)	
yes	15 (4.25)	6 (3.08)	9 (5.70)		14 (11.67)	2 (3.39)	12 (19.67)	
Spiculation, No. (%)				0.237				0.727
no	136 (38.53)	81 (41.54)	55 (34.81)		54 (45.00)	28 (47.46)	26 (42.62)	
yes	217 (61.47)	114 (58.46)	103 (65.19)		66 (55.00)	31 (52.54)	35 (57.38)	
Lobulation, No. (%)				0.297				0.747
no	98 (27.76)	59 (30.26)	39 (24.68)		80 (66.67)	38 (64.41)	42 (68.85)	
yes	255 (72.24)	136 (69.74)	119 (75.32)		40 (33.33)	21 (35.59)	19 (31.15)	
Vascular convergence, No. (%)				<0.001				0.532
no	88 (24.93)	70 (35.90)	18 (11.39)		77 (64.17)	40 (67.80)	37 (60.66)	
yes	265 (75.07)	125 (64.10)	140 (88.61)		43 (35.83)	19 (32.20)	24 (39.34)	
Pleural traction, No. (%)				0.685				0.740
no	193 (54.67)	109 (55.90)	84 (53.16)		46 (38.33)	24 (40.68)	22 (36.07)	
yes	160 (45.33)	86 (44.10)	74 (46.84)		74 (61.67)	35 (59.32)	39 (63.93)	
Pleural indentation, No. (%)				0.86				0.826
no	253 (71.67)	141 (72.31)	112 (70.89)		49 (40.83)	23 (38.98)	26 (42.62)	
yes	100 (28.33)	54 (27.69)	46 (29.11)		71 (59.17)	36 (61.02)	35 (57.38)	
Air bronchogram, No. (%)				0.142				0.881
no	271 (76.77)	156 (80.00)	115 (72.78)		98 (81.67)	49 (83.05)	49 (80.33)	
yes	82 (23.23)	39 (20.00)	43 (27.22)		22 (18.33)	10 (16.95)	12 (19.67)	
Vacuole, No. (%)				0.841				0.708
no	231 (65.44)	129 (66.15)	102 (64.56)		97 (80.83)	49 (83.05)	48 (78.69)	
yes	122 (34.56)	66 (33.85)	56 (35.44)		23 (19.17)	10 (16.95)	13 (21.31)	
Tumor location, No. (%)				0.128				0.756
LUL	94 (26.63)	53 (27.18)	41 (25.95)		36 (30.00)	15 (25.42)	21 (34.43)	
LLL	78 (22.10)	35 (17.95)	43 (27.22)		22 (18.33)	12 (20.34)	10 (16.39)	
RUL	103 (29.18)	64 (32.82)	39 (24.68)		26 (21.67)	15 (25.42)	11 (18.03)	
RML	19 (5.38)	8 (4.10)	11 (6.96)		4 (3.33)	2 (3.39)	2 (3.28)	
RLL	59 (16.71)	35 (17.95)	24 (15.19)		32 (26.67)	15 (25.42)	17 (27.87)	
Operative mode, No. (%)				<0.001				0.900
wedge resection	50 (14.16)	40 (20.51)	10 (6.33)		17 (14.17)	9 (15.25)	8 (13.11)	
sublobar resection	47 (13.31)	29 (14.87)	18 (11.39)		7 (5.83)	3 (5.08)	4 (6.56)	
lobectomy	256 (72.52)	126 (64.62)	130 (82.28)		96 (80.00)	47 (79.66)	49 (80.33)	

SD, Standard deviation; STAS, Spread through air space; WBC, white blood cell; RLL, Lower Lobe; FEV1, forced expiratory volume in 1 second; DLCO, diffusing capacity of the carbon monoxide; WBC, white blood cell; NLR, neutrophil to lymphocyte ratio; MLR, monocyte to lymphocyte ratio; PLR, platelet to lymphocyte ratio; CA125, carbohydrate antigen 125; AFP, alpha-Fetoprotein; CEA, carcinoembryonic antigen; NSE, Neuron-Specific Enolase; cyfra21-1, cytokeratin 19 Fragment 21-1; proGRP, pro-Gastrin-Releasing Peptide.

### Selection of variables

**Figure 2:**
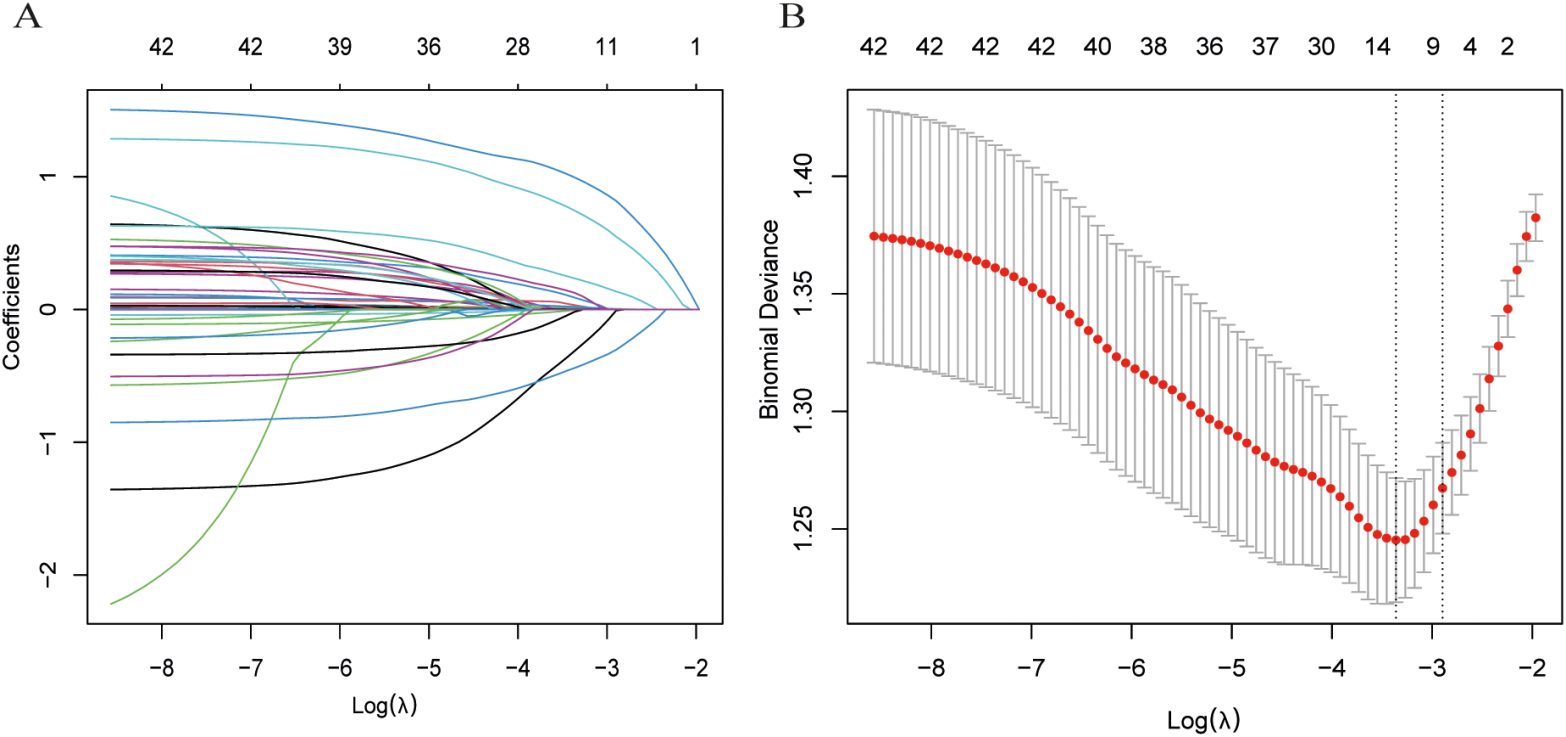
Process of selecting variables through LASSO regression. **(A)** LASSO coefficient profiles across a sequence of log(λ) values. The optimal penalty parameter λ was determined via ten-fold cross-validation. **(B)** Validation of the optimal λ, with dotted vertical lines indicating the chosen value. Seven variables with nonzero coefficients were selected by λ. min.

We first employed mRMR for initial variable screening to maximize the correlation between features while minimizing inter-feature redundancy, followed by LASSO to identify key STAS-related variables (**Figures 2A, B**). The selected predictive variables were: CTR, age, smoking history, AFP, CEA, proGRP, and vascular convergence.

### Model construction and validation

Using the 7 selected variables, seven machine learning algorithms—logistic regression, random forest, SVM, XGBoost, AdaBoost, LightGBM, and CatBoost—were employed to construct and validate predictive models.

the AdaBoost model demonstrated the highest discriminative ability, with an area under the curve (AUC) of 0.963 (95% CI: 0.947–0.980), along with a sensitivity of 0.886, specificity of 0.908, positive predictive value (PPV) of 0.886, and negative predictive value (NPV) of 0.907. However, the Hosmer-Lemeshow test indicated poor calibration (P = 1.33 × 10^-15^), suggesting a significant discrepancy between the predicted probabilities and the observed outcomes.

**Figure 3:**
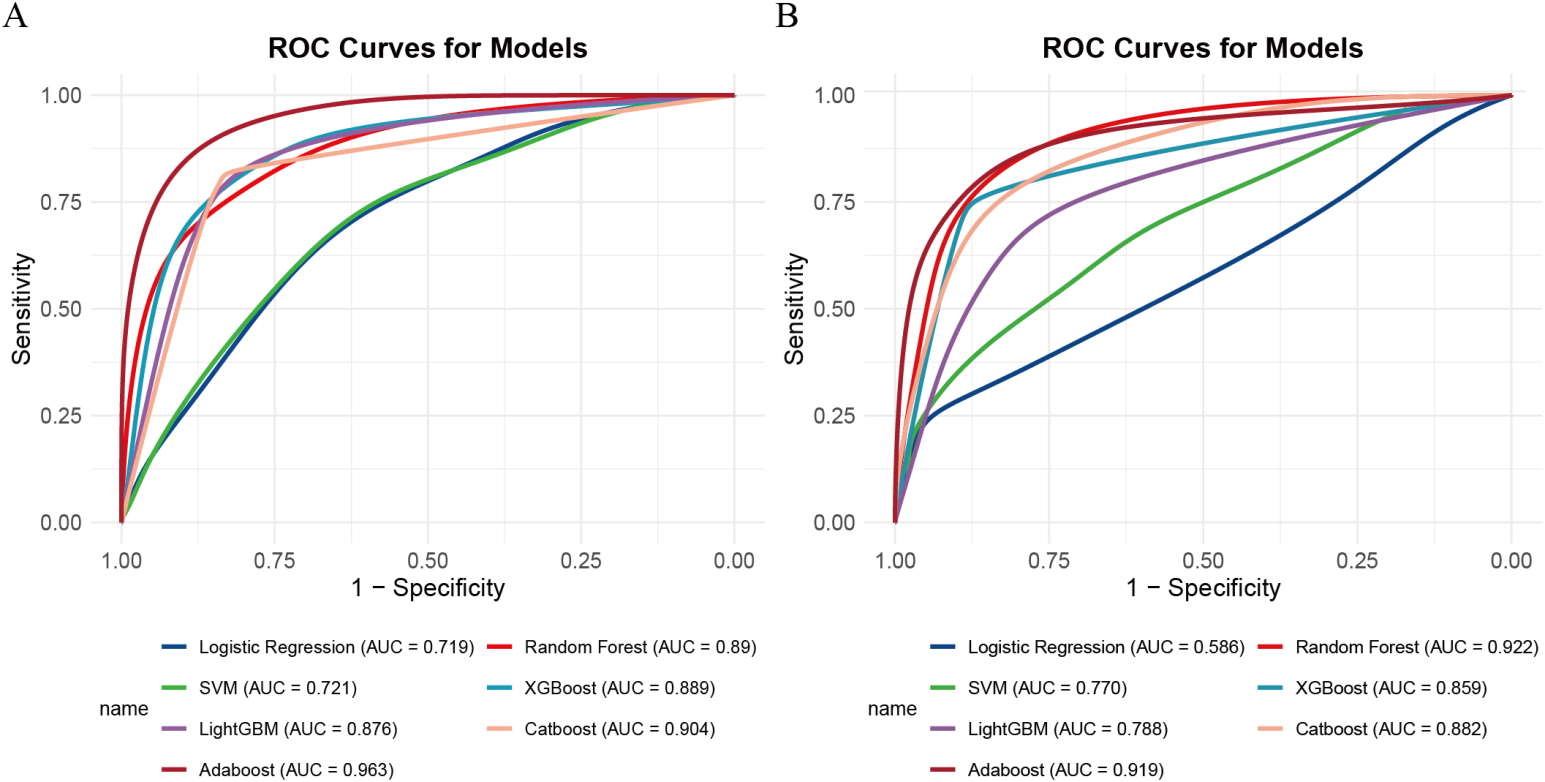
ROC curves for seven machine learning models. **(A)** Training set, **(B)** validation set. ROC, receiver operating characteristic.

**Figure 4:**
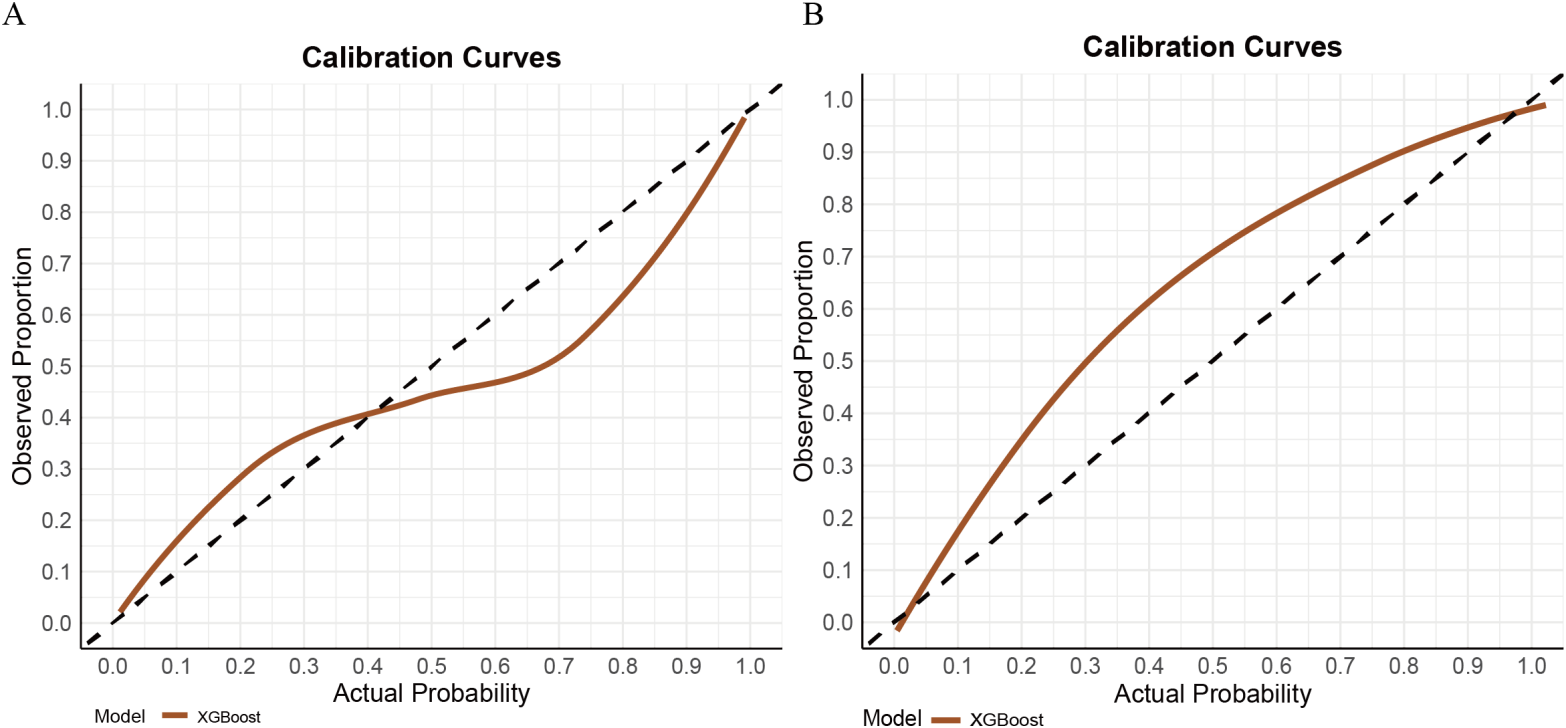
Calibration curves for XGBoost model in the train set **(A)** and validation **(B)** set.

In contrast, the XGBoost model exhibited strong and more balanced performance across both the training and validation sets. In the training set, XGBoost achieved an AUC of 0.889 (95% CI: 0.852–0.926), with a sensitivity of 0.810, specificity of 0.805, PPV of 0.701, and NPV of 0.840. In the validation cohort, the model demonstrated an AUC of 0.856 (95% CI: 0.789–0.928), with sensitivity, specificity, PPV, and NPV of 0.738, 0.881, 0.865, and 0.765, respectively (see **Figure 3**). Its calibration curve demonstrated close agreement between observed and predicted risks (**Figure 4**), indicating good calibration. A comprehensive summary of all models is provided in **Table 2**. The calibration plot demonstrated close agreement between observed and predicted outcomes, indicating good predictive accuracy of the model. (see **Figure 4**). A comprehensive performance summary for each model is presented in **Table 2**.

**Table 2:** Performance of seven models.

Model	Group	AUC (95%CI)	Accuracy	Specificity	Sensitivity	NPV	PPV	F1
logistic regression	train	0.719 ( 0.667 - 0.772)	0.677	0.703	0.646	0.710	0.638	0.642
	validation	0.586 (0.483 - 0.689)	0.567	0.814	0.328	0.541	0.645	0.435
random forest	train	0.890 ( 0.857 - 0.923)	0.793	0.821	0.759	0.805	0.774	0.767
	validation	0.922 (0.886-0.97)	0.867	0.881	0.852	0.865	0.881	0.867
SVM	train	0.721 ( 0.667 - 0.774)	0.669	0.672	0.665	0.726	0.621	0.642
	validation	0.770 ( 0.686 - 0.853)	0.633	0.898	0.377	0.582	0.793	0.511
XGBoost	train	0.889 ( 0.852- 0.926)	0.807	0.805	0.81	0.840	0.771	0.79
	validation	0.859 ( 0.789 - 0.928)	0.808	0.881	0.738	0.765	0.865	0.796
AdaBoost	train	0.963 ( 0.947 - 0.980)	0.898	0.908	0.886	0.907	0.886	0.886
	validation	0.919 ( 0.866 - 0.972)	0.817	0.915	0.721	0.761	0.898	0.8
LightGBM	train	0.876 ( 0.838 - 0.913 )	0.822	0.831	0.81	0.853	0.795	0.803
	validation	0.788 ( 0.705 - 0.872 )	0.725	0.814	0.639	0.685	0.78	0.703
CatBoost	train	0.904 ( 0.872 - 0.935 )	0.819	0.836	0.797	0.836	0.797	0.797
	validation	0.882 ( 0.8226 - 0.942 )	0.792	0.864	0.721	0.750	0.846	0.779

CI, Confidence interval; AUC, Area under the receiver operating characteristics curve; F1, F1 score; SVM, support vector machines; XGBoost, Extreme Gradient Boosting; AdaBoost, adaptive boosting; LightGBM, light gradient boosting machine; CatBoost, category boosting; NPV, negative predictive value; PPV, positive predictive value.

**Figure 5:**
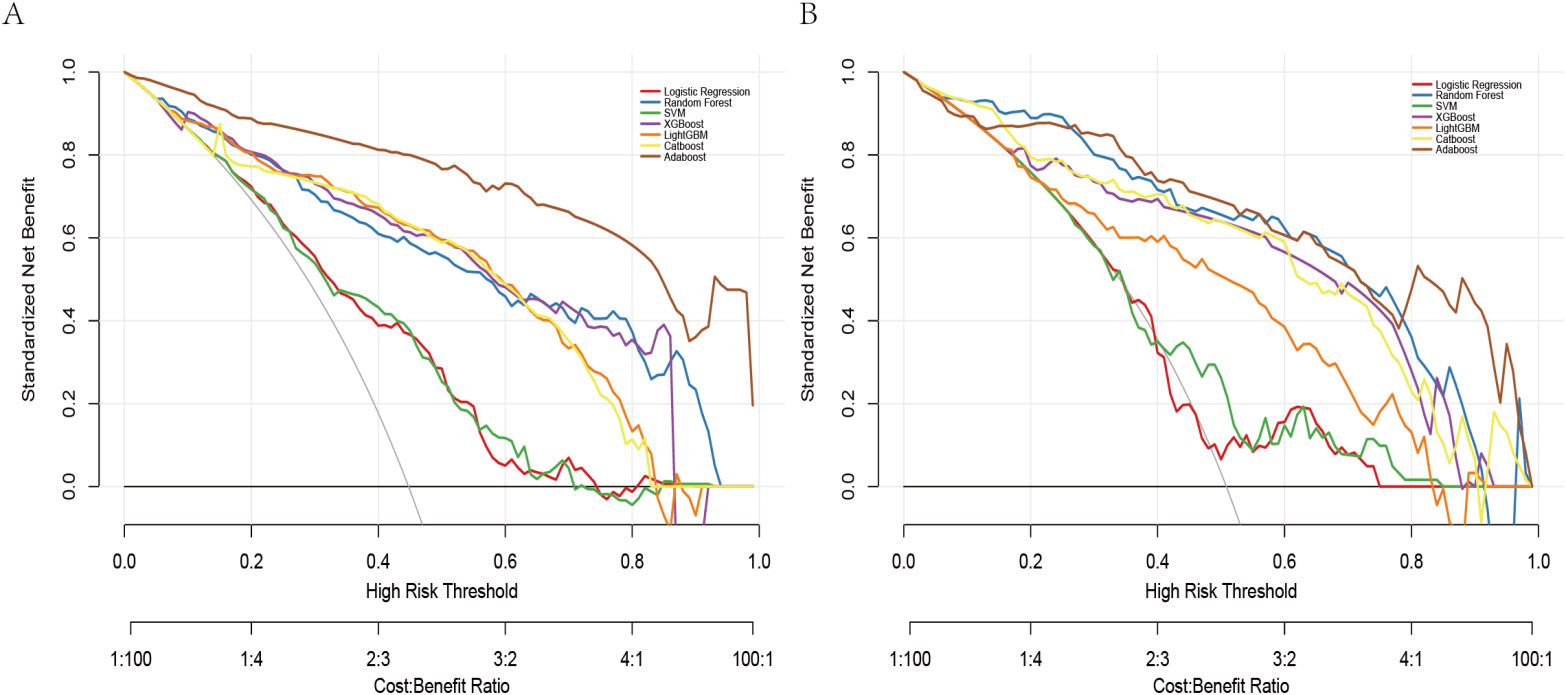
Decision curves for seven machine learning models: **(A)** training set, **(B)** validation set.

The DCA revealed all seven models consistently provided a net benefit greater than 0 across a range of threshold probabilities (0.0 to 0.8), suggesting their potential clinical utility in guiding STAS prediction decisions (**Figure 5**). The net benefit rate for all models remained above 0 in both the training and validation sets. The XGBoost model maintained a favorable net benefit within the clinically relevant cost-benefit ratio range (1:4 to 4:1), highlighting its robustness and practical value in clinical settings. To clarify threshold selection and clinical use, we focused on 0.20–0.80 as a prespecified, clinically plausible range based on the accepted trade-off between missing STAS and unnecessary escalation. Within this range—particularly around 0.30–0.50—XGBoost offers the most consistent net benefit, supporting model-guided escalation when the consequence of missing STAS is considered high, whereas higher thresholds (≥0.60) help limit overtreatment.

Taken together, while AdaBoost delivered the highest AUC, however, its calibration performance was markedly unsatisfactory, as indicated by the Hosmer–Lemeshow test (P = 1.33×10^-15^) and the corresponding calibration curves (see **Supplementary Figure 1**), which revealed a considerable discrepancy between predicted and observed outcomes. In contrast, XGBoost provided a more balanced performance: it maintained strong discrimination while demonstrating good calibration, with predicted risks well aligned with actual probabilities (see **Figure 3**). From a clinical perspective, accurate probability estimates are crucial for risk stratification and surgical decision-making, where over- or underestimation of risk may lead to inappropriate treatment choices. Therefore, despite its slightly lower AUC, XGBoost was chosen as the preferred model.

### Model explanation

**Figure 6:**
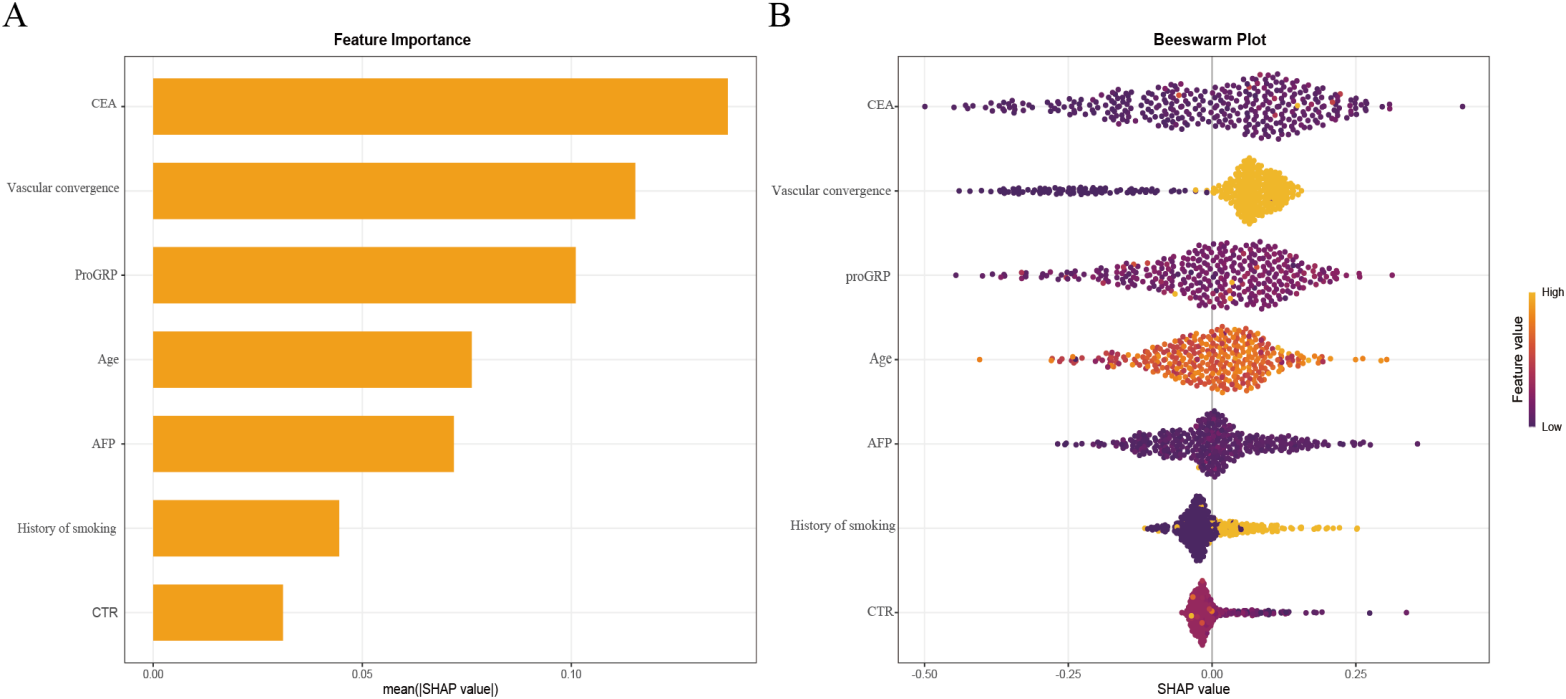
XGBoost model interpretability using SHAP.SHAP summary bar plot ranking feature importance **(A)**. SHAP summary dot plot illustrating feature contributions **(B)**. Each dot represents an individual patient’s SHAP value for a given feature, with color intensity indicating the feature’s actual value. Higher SHAP values reflect an increased probability of STAS positivity.

To enhance clinical interpretability, we used SHAP to quantify both the direction and magnitude of each feature’s contribution to the final XGBoost model’s predictions. **Figure 6A** summarizes global importance as the mean absolute SHAP value for each feature, with CEA contributing the most overall, followed by vascular convergence, proGRP, age, AFP, smoking history, and CTR. In the beeswarm plot (**Figure 6B**), features are ordered by mean absolute SHAP value (global importance). Horizontal position reflects the SHAP value for each case (positive values increase the predicted probability of STAS; negative values decrease it), while color encodes the feature value (yellow = higher value, purple = lower value). Consistent with the biological rationale, higher CEA values (yellow points) cluster toward positive SHAP values, indicating that elevated CEA is associated with an increased predicted risk of STAS. A similar positive directionality is observed for vascular convergence and CTR (higher values tend to push predictions toward higher STAS probability). proGRP and AFP exhibit modest positive contributions at higher values, and smoking history (ever vs. never) shifts predictions toward higher risk. Age shows a smaller but directionally consistent effect.

To facilitate clinical utility, a final prediction nomogram was constructed using the seven predictive variables (CEA, vascular convergence, proGRP, age, AFP, smoking history, CTR) and implemented in a web-based application for clinical use. The web application is accessible online at: https://cuncun.shinyapps.io/DynNomapp/.

## Discussion

This study presents a retrospective analysis of clinical features associated with STAS in stage I lung adenocarcinoma, utilizing seven risk factors (CEA, vascular convergence, proGRP, age, AFP, smoking history, and CTR) to develop predictive models. We compared seven machine learning models, with AdaBoost demonstrating the best diagnostic performance. However, XGBoost exhibited superior discriminatory power and predictive accuracy, We employed the SHAP function to visualize model interpretability, thereby enhancing the model’s transparency.

Our study has several advantages (1). Multi-center design: This is a multi-center retrospective study leveraging preoperative clinical indicators to predict STAS in stage I lung adenocarcinoma, aiming to develop a predictive model for assessing STAS risk.​​ (2) Clinical variables: Unlike radiomics-based studies, the clinical variables (27, 28), used in this model are easily accessible, significantly improving the model’s generalizability. Additionally, we developed a web-based nomogram, which facilitates practical use in clinical settings.​​ ​​ (3) Superior interpretability: Compared to other ML models, our nomogram shows similar diagnostic performance but offers superior interpretability and operational ease.

STAS has emerged as a critical pathological feature linked to local recurrence and poor prognosis in lung cancer (10, 29, 30). It is a powerful independent predictor of recurrence and prognosis in stage I lung adenocarcinoma. STAS-positive patients exhibit significantly worse postoperative outcomes than STAS-negative patients in stage IA, with prognosis approaching that of stage IB patients (31, 32). STAS also predicts recurrence and prognosis in stage I lung squamous cell carcinoma, although this association does not extend to stages II–III (8). Radiologically, the presence of solid components correlates with higher STAS risk, with a threefold increase in STAS risk for every 1% increase in the solid component of the tumor (33).

Surgical outcomes are significantly influenced by STAS. In T1N0M0 lung adenocarcinoma, patients with STAS who undergo sublobar resection have higher recurrence rates and lung cancer–specific mortality than those treated with lobectomy (34). Moreover, among sublobar approaches, wedge resection is associated with inferior OS and RFS compared with segmentectomy or lobectomy (11). Therefore, accurate preoperative identification of STAS-positive patients is crucial for surgical decision-making—particularly when considering sublobar resection—as failure to recognize STAS preoperatively may lead to undertreatment and a substantially increased risk of recurrence. Patients at high predicted risk may be considered for lobectomy to mitigate recurrence risk.

Our study identified several predictive factors, including baseline characteristics, CT imaging features and tumor markers, Smoking, the most significant risk factor for lung cancer, has been linked to increased STAS risk, particularly in older patients (10, 32). However, the exact relationship between smoking, age, and STAS positivity remains unclear and warrants further investigation.

Tumor markers, including CEA, proGRP, AFP reflect tumor biology and systemic disease burden (35). CEA, a glycoprotein associated with cell adhesion, is typically absent in healthy adult blood (36). Our feature interpretability analysis revealed CEA as the most significant factor in the XGBoost model, which is consistent with findings from other studies (37, 38). High CEA expression can promote epithelial-mesenchymal transition (EMT) by modulating various signaling molecules within the EMT pathway. During EMT, tumor cells lose epithelial adhesion markers and gain mesenchymal markers, enhancing motility and invasiveness, which in turn increases STAS likelihood (31, 39).

ProGRP plays a vital role in diagnosing and subtyping lung cancer (40), particularly in small cell lung cancer(SCLC). It has been widely applied as a biomarker for SCLC diagnosis, monitoring, and evaluation of treatment response (41, 42), and is also considered an effective marker for diagnosing lung neuroendocrine neoplasms (43). Recent studies further suggest that ProGRP, when combined with artificial intelligence approaches, can accurately predict lung cancer risk (44). Nevertheless, the role of ProGRP in lung adenocarcinoma remains insufficiently understood, and further research is warranted to clarify its potential diagnostic and prognostic value.

AFP is a glycoprotein originally identified as the first oncoprotein and is now widely used as a biomarker in hepatocellular carcinoma screening (45, 46), Elevated serum AFP levels have also been reported in some patients with primary lung cancer (47, 48), and extremely high concentrations are a distinguishing feature of hepatoid adenocarcinoma of the lung (49), However, the intrinsic relationship between AFP and lung adenocarcinoma remains poorly understood and warrants further investigation.

Vascular convergence has been identified as a strong indicator of STAS, appearing frequently in STAS-positive patients (50, 51).

The aggressiveness of lung cancer is also linked to the proportion of solid tumor components observed on CT, a higher solid component indicates a more significant, CTR have a positive correlation with STAS (32, 52) and as the most accurate CT characteristic for forecasting STAS in lung adenocarcinomas measuring ≤2 cm (53). Our research shows that an increase in solid components is an independent predictor of STAS, significantly heightening the risk, consistent with previous studies.

In this study, we utilized clinical baseline characteristics, imaging characteristics and tumor markers to develop various machine learning models to preoperatively predict the presence of STAS preoperatively. the XGBoost model, which effectively manages high-dimensional data and complex interactions, showed superior performance, with the predicted values aligning closely with actual results. The SHAP algorithm was used to enhance model interpretability, making the results more accessible to clinicians.

Previous studies based on CT radiomics models have faced challenges due to the low incidence of STAS and the typically single-center design of such studies, limiting their generalizability. Multi-center studies have also struggled with robustness (28, 54).

Additionally, radiomics models often suffer from a lack of interpretability, creating a “black box” effect that reduces clinical confidence. In contrast, the clinical variables in our model are derived from preoperative data and CT images, which are easily accessible. The use of the SHAP algorithm further enhances interpretability, and the model can be accessed through a web-based platform, improving clinical applicability.

Nonetheless, this study has several limitations. First, the retrospective design introduces potential selection bias, highlighting the need for prospective validation. Second, although external validation was performed, it was derived from a single-center cohort, which limits the generalizability of the findings. Third, the relatively small sample size raises concerns about potential overfitting of the model, and the lack of long-term follow-up further restricts the strength of the conclusions. In addition, pure GGO nodules and patients with multiple nodules were excluded; future investigations should develop strategies to better evaluate STAS in these subgroups. Overall, larger multi-center prospective studies with extended follow-up are required to confirm and extend our findings. However, the web-based tool developed in this study has not yet undergone prospective, multi-center validation or formal clinical impact assessment, and thus its clinical applicability remains preliminary. At this stage, it should be regarded as a research prototype rather than a tool to guide individual patient care.

The predictive models based on XGBoost regression demonstrated significant preoperative predictive accuracy for STAS in stage I LUAD solid and part-solid nodules. The application of SHAP analysis augmented the model’s interpretability by establishing associations between predictions and relevant clinical variables, thereby enhancing its clinical applicability. This interpretable model offers a promising tool for personalized preoperative surgical planning and tailored postoperative management.

## Data Availability

The raw data supporting the conclusions of this article will be made available by the authors, without undue reservation.

## Supplementary material

Supplementary Figure 1 Calibration curves of seven machine learning models in the training set **(A)** and validation set **(B)**. Closer alignment of the curves with the diagonal line indicates better agreement between predicted and observed probabilities.
