# Supplementary figures and images for: Development and validation of machine learning models for predicting STAS in stage I lung adenocarcinoma with part-solid and solid nodules: a two-center study
Source: Front Oncol. 2025 Oct 29;15:1682633. doi: 10.3389/fonc.2025.1682633 (PMC12605206; doi:10.3389/fonc.2025.1682633)

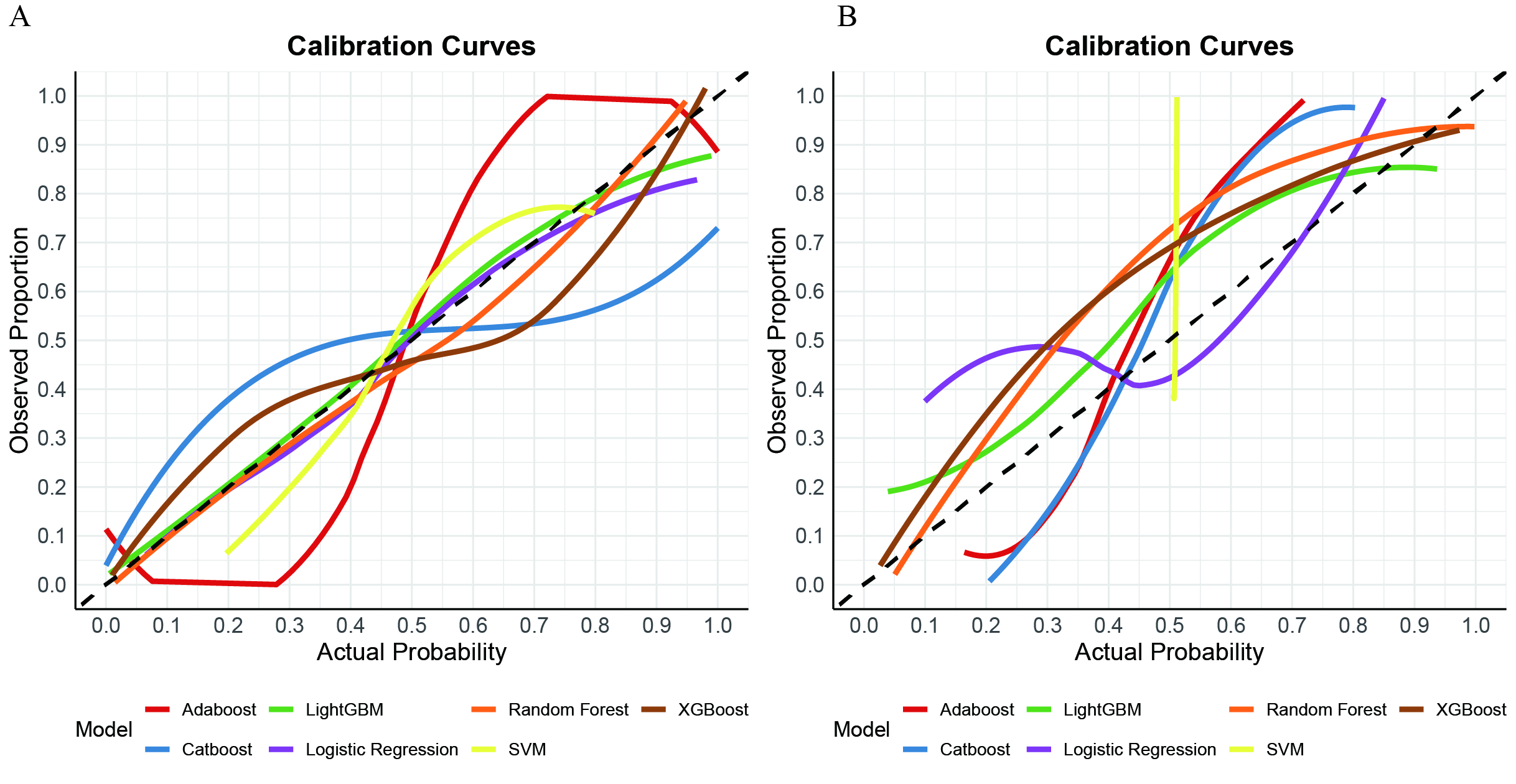

Supplement: Supplementary Figure 1 — Calibration curves of seven machine learning models in the training set (A) and validation set (B). Closer alignment of the curves with the diagonal line indicates better agreement between predicted and observed probabilities. [file Image1.tif]
